# Species-Specific Immunodetection of an *Entamoeba histolytica* Cyst Wall Protein

**DOI:** 10.1371/journal.pntd.0004697

**Published:** 2016-05-06

**Authors:** Lauren J. Spadafora, Moira R. Kearney, Abdullah Siddique, Ibne K. Ali, Carol A. Gilchrist, Tuhinur Arju, Benjamin Hoffstrom, Felicia K. Nguyen, William A. Petri, Rashidul Haque, Gerard A. Cangelosi

**Affiliations:** 1 Department of Global Health, University of Washington, Seattle, Washington, United States of America; 2 Department of Environmental and Occupational Health Sciences, University of Washington, Seattle, Washington, United States of America; 3 International Centre for Diarrhoeal Disease Research, Bangladesh, Dhaka, Bangladesh; 4 Department of Medicine, School of Medicine, University of Virginia, Charlottesville, Virginia, United States of America; 5 Antibody Technology Core, Fred Hutchinson Cancer Research Center, Seattle, Washington, United States of America; Jawaharlal Nehru University, INDIA

## Abstract

*Entamoeba histolytica* causes intestinal disease in endemic settings throughout the world. Diagnosis of *E*. *histolytica* infection would be improved by the identification of biomarkers that are expressed by cysts of *E*. *histolytica*, but not by cysts of closely related commensal species of *Entamoeba*. Herein, we describe two novel monoclonal antibodies (1A4 and 1D3) produced against a spacer region of the *E*. *histolytica* Jacob2 lectin, an outer cyst wall protein. These reagents demonstrated no cross-reaction to *E*. *dispar* recombinant antigen and low picomolar molecular detection limits when paired in ELISA sandwich assays. In an immunofluorescence microscopy assay, the α-Jacob2 murine antibodies labeled cysts of three xenically cultured *E*. *histolytica* isolates but did not label cysts of three *E*. *bangladeshi* isolates. Monoclonal antibody 1A4 did not cross-react with xenic cultures of three *E*. *dispar* isolates, demonstrating specificity to *E*. *histolytica*, while monoclonal antibody 1D3 cross-reacted with two out of three *E*. *dispar* isolates. Both antibodies labeled cysts in formalin-fixed slides, a potential logistical advantage in some settings. The monoclonal antibody 1A4 was also used in an immunofluorescence microscopy assay with formalin-fixed stool specimens. Seven out of ten ELISA-positive stool specimens exhibited 1A4-labeled cyst-like objects, compared to one out of seven ELISA-negative specimens. These results demonstrate that antibodies generated against the flexible spacer of *E*. *histolytica* Jacob2 lectin recognize and bind to Jacob2 protein in whole cysts and are capable of differentiating *Entamoeba* species in fixed specimens. Thus, Jacob2 is a promising biomarker for use in diagnosing *E*. *histolytica* infection.

## Author Summary

*Entamoeba histolytica* is a prevalent human parasite requiring sensitive and specific detection. Assays available for *Entamoeba* detection utilize antibodies to detect parasite protein in stool, and they distinguish *E*. *histolytica* from nonpathogenic commensal amoeba. However, these tests have exhibited suboptimal sensitivity in some studies. This may have occurred because the tests do not detect the cyst stage of the parasite, which is more prevalent and stable in stool. Moreover, they cannot be used on formalin-fixed material, which creates logistical problems in some settings. Here, we have generated antibodies against a region of an *E*. *histolytica* cyst surface protein that has a unique sequence relative to nonpathogenic amoeba. One of these antibodies bound to *E*. *histolytica* cysts in fixed, xenic cultures without cross-reacting to nonpathogenic *E*. *dispar* and *E*. *bangladeshi*, while another antibody bound to both *E*. *histolytica* and *E*. *dispar*. The antibody that selectively bound to *E*. *histolytica* cysts also labeled cyst-like objects in fixed stool specimens from *E*. *histolytica-*positive patients. As they detected fixed organisms, these antibodies could ultimately make specimen collection and processing for *Entamoeba* diagnosis a simpler process. Additionally, this study indicates that the antibodies’ cyst protein target may be a useful biomarker for *E*. *histolytica* detection.

## Introduction

*Entamoeba histolytica* is a protozoan parasite that causes an estimated 30–50 million cases of illness and kills 100,000 people annually [1,2]. It has a dual-stage life cycle, consisting of motile trophozoites that colonize and invade the colonic epithelium and of robust, chitinaceous cysts that enter and exit the human body via fecal-oral transmission [3,4]. Infection is often asymptomatic, or it can lead to clinical manifestations that include dysentery, colitis, or extraintestinal abscesses [3–5]. Symptoms often resemble other enteric diseases caused by bacteria and viruses, as well as inflammatory bowel disease [3,4]. Control of this organism is particularly important for young children in endemic regions, whose nutrition, growth, and development are negatively impacted by enteric infections and repeated diarrheal episodes [6–8].

Detecting *E*. *histolytica* can be challenging due to the numerous commensal amoeba that colonize humans, some of which look morphologically identical to the pathogen [9–11]. Much work has been undertaken to identify and characterize superior biomarkers of infection in stool and of invasive disease in serum and abscess fluid. One such marker is the galactose/N-acetyl galactosamine (Gal/GalNAc) lectin, an adhesion factor that is important to *E*. *histolytica* trophozoite invasion [12]. This protein is the target of two antigen capture assays that have been widely and successfully used to specifically detect *E*. *histolytica* infections in fresh stool or liver abscesses [13–15]. However, these tests are unable to detect the cyst stage of the parasite, and they cannot be applied to formalin-fixed specimens [9,15,16]. The instability of shed trophozoites in unfixed specimens necessitates prompt transport and testing, a significant logistical limitation in some settings [2,9,15,16]. These limitations may have an impact on *E*. *histolytica* diagnosis, as one of the tests was found to be at best 79% sensitive relative to the more sensitive qPCR method [17].

A cyst wall lectin with diagnostic potential, named Jacob2, was described recently [18]. It is one of a few proteins known to be expressed only in the *Entamoeba* cyst stage [19–21]. In the “wattle and daub” model of *Entamoeba* encystation, the Jacob2 protein first appears in intracellular vesicles and is secreted through the plasma membrane [19,22]. Then, it is tethered by the Gal/GalNAc lectin and binds to chitin to form the cyst wall via three, chitin-binding domains [18–20,22,23]. Between these domains is a flexible, serine-rich spacer sequence with an amino acid sequence dissimilar between *E*. *histolytica* and commensal non-pathogenic species *E*. *dispar* [18,19]. This sequence was also noted to be polymorphic between evaluated *E*. *histolytica* strains [18,19]. Nevertheless, Jacob2 and other cell wall components could potentially be utilized to distinguish *E*. *histolytica* from similar non-pathogenic species such as *E*. *dispar*.

Here, we describe the generation of murine monoclonal antibodies against the variable spacer region of the *E*. *histolytica* Jacob2 protein. These reagents demonstrated excellent analytical sensitivity in a sandwich ELISA. More importantly, one of them bound *E*. *histolytica* cysts in xenic culture without cross-reacting to xenic isolates of the commensal species *E*. *dispar* and of the recently discovered species, *Entamoeba bangladeshi*, which was first described in 2012 based on genotypic and morphotypic criteria [11]. This same monoclonal antibody was effective at labeling cyst-like objects in formalin-fixed stool specimens. This study supports the feasibility of using the Jacob2 spacer region as an *E*. *histolytica-*specific cyst biomarker.

## Methods

### Ethics Statement

Stool specimens were collected from subjects in Dhaka, Bangladesh, with approval from the Ethics Review Committee of the International Centre for Diarrheal Disease Research, Bangladesh (ICDDR,B) and the Institutional Review Board (IRB) of the University of Washington. Informed, written consent was obtained from patients or from guardians of subjects ages 1–17, and assent was obtained from subjects ages 8–17. Monoclonal antibody production was approved by the Fred Hutchinson Cancer Research Center (FHCRC) Institutional Animal Care and Use Committee (IACUC). Animal care protocols at FHCRC follow all federal guidelines, including the Public Health Service (PHS) Policy on Human Care and Use of Laboratory Animals, the United States Department of Agriculture (USDA) Animal Welfare Act, Code of Federal Regulations, Title 9, Chapter 1, Subchapter A—Animal Welfare, and the terms of the facility's PHS Animal Welfare Assurance.

### Bioinformatics

The Jacob2 sequences for *E*. *histolytica* strain HM-1:IMSS (EHI_044500) and for *E*. *dispar* strain SAW760 (EDI_246160) were stored on Geneious version 6.0.3 and aligned utilizing the ClustalW BLOSUM cost matrix, with a gap open cost of 10 and a gap extend cost of 0.1.

### Recombinant Protein Production

All cloning and expression reagents were obtained from Life Technologies (Carlsbad, CA) unless otherwise noted. Residues 159–481 of the *E*. *histolytica* strain HM-1:IMSS Jacob2 protein (EhJacob) were codon-optimized and TOPO-TA-cloned into the pET SUMO vector [24]. Residues 212–560 of the *E*. *dispar* strain SAW670 (EdJacob) were cloned into the Gateway pDEST17 vector with a N-terminal 6xHis tag. The constructs were expressed in BL21(DE3) *Escherichia coli* cells, and protein was purified via Ni-NTA resin (Qiagen, Hilden, Germany). The SUMO tag on EhJacob was cleaved with SUMO protease (Lifesensors, Malvern, PA) and removed by Ni-NTA resin according to kit instructions.

### Murine Monoclonal Antibody Production

Murine monoclonal antibodies were generated by the Antibody Technology Core at the Fred Hutchinson Cancer Research Center (Seattle, WA). EhJacob was dialyzed into 1x PBS and combined with three additional *E*. *histolytica* recombinant antigens (EHI_101240, EHI_070730, and EHI_104630) for immunization. The multiplex antigen formulation was used to generate antibodies for multiple investigations including this one. Three sets of splenic cell fusions were performed: the first was from high-titer mice and was screened for specificity via an indirect ELISA, while the second and third were from lower-titer mice and were first selected for IgG secretion with a ClonePixII system (Molecular Devices, Sunnyvale, CA), followed by target specific indirect ELISA screening. The isotypes were determined by indirect ELISA assays.

### Screening of Monoclonal Antibodies by Indirect and Matrix Sandwich ELISAs

IgG3 antibodies are not frequently found in outputs and thus were not screened, and IgM were not screened due to their typically low affinities and specificities. The 48 IgG1 and 16 IgG2 antibodies with highest binding activity (OD >1.0) were screened as pairs in 48 x 16 matrix sandwich ELISAs. First, anti-IgG1 antibody (0.5 μg/mL, Life Technologies A10538) was coated overnight at 4°C to high binding, 384-well plates (Greiner Bio-One, Kremsmünster, Austria). Next, the following materials were incubated for 1 hour at room temperature with subsequent three rounds of washing with PBS + 0.05% Tween 20 (PBS-T): 1) 25 μL of 1:8 IgG1 supernatants; 2) 125 ng/mL EhJacob (with SUMO tag) or EdJacob antigen; 3) 25 μL of 1:8 IgG2 supernatants; and 4) 1:2000 HRP-conjugated anti-IgG2a or–IgG2b antibodies (Life Technologies A10685 and M32507). Wells were developed with ABTS (Kirkegaard & Perry Laboratories, Gaithersburg, MD) for 15 minutes and read at 405 nm, with threshold set at an optical density of 0.7.

The specificities of top antibodies were checked once again by indirect ELISA, this time incorporating two non-cognate antigens, one of which had a SUMO tag. Wells of a Nunc Maxisorp flat bottom plate (Nunc, Rochester, NY) were coated with 200 ng of antigen diluted in 50 mM bicarbonate buffer, pH 9.6 (Sigma Aldrich, St. Louis, MO) overnight at 4°C. After the plate was washed three times in PBS-T and blocked with Starting Block (Life Technologies) for 1 hour at room temperature, wells incubated with selected hybridoma supernatants diluted 1:5–1:20 in Starting Block for 1 hour at room temperature. The plate was washed again and incubated with 1:1000 of goat anti-mouse HRP (H+L) (Bio-Rad Laboratories, Inc., Heracles, CA) for 1 hour at room temperature. One-step Ultra TMB substrate (Life Technologies) was added after four additional washes with PBS-T, and the reaction was stopped with 2 N hydrochloric acid upon development of color. The microplates were read at 450 nm.

### Sandwich ELISA with Purified Antibody

Wells in flat-bottom Maxisorp microplates were coated with 0.15 μg of purified monoclonal antibody 1A4 diluted in 50 mM bicarbonate buffer, pH 9.6, overnight at 4°C. Simultaneously, an additional set of wells were coated with blank buffer to serve as a control with no capture antibody. After three washes in PBS-T, wells were blocked with 1% BSA in PBS-T for 90 minutes at room temperature. Next, a serial titration of SUMO-EhJacob, ranging 16.4 pg to 17.1 ng, was plated and incubated for 60 minutes at room temperature, followed by three washes in PBS-T. Monoclonal antibody 1D3 (1.2 μg/mL) and 125 ng/mL HRP-conjugated goat anti-mouse IgG2a (#ab97245, Abcam, Cambridge, MA) incubated for 1 hour at RT in succession, followed by three and four washes in PBS-T respectively. Finally, wells were developed with 100 μL of 1-Step Ultra TMB solution for 60–80 seconds and stopped with 2 N hydrochloric acid. The microplates were read at 450 nm.

### Xenic Entamoeba spp. Culture in Robinsons Diphasic Media

Stool specimens were obtained from children enrolled in an ongoing community-based prospective cohort study of enteric infections [25]. Diarrheal and monthly surveillance stools were examined by microscopy, and a 50 mg aliquot of any prospective *Entamoeba* positive feces was inoculated into a 7 mL glass McCartney bottle containing a sterile agar slant and a liquid overlay of Robinson’s media, bacto-peptone, erythromycin, and 10 mg of rice starch [26,27]. Bottles were incubated at 37°C for 24 hours. Next, a drop of culture from the fecal-starch layer was drawn up and examined by microscopy. If no amoeba were found, additional rice starch was added to the bottle, and the culture was incubated for an additional 48 hours before re-examination. If amoeba were identified, the culture was passaged every 48 hours by inoculation of 0.1 mL of fecal-starch layer into a fresh bottle of media. *Entamoeba dispar* and *E*. *bangladeshi* isolates were identified by PCR, whereas *E*. *histolytica isolates* were confirmed by both PCR and ELISA (Techlab, Blacksburg, VA).

### Immunofluorescence Assay on Xenic Cultures

Xenic cultures were briefly checked for the presence of *Entamoeba*-like organisms by light microscopy prior to slide preparation. Smears were first air dried onto Merifluor treated slides (Meridian Bioscience, Inc., Cincinnati, OH) and then fixed in 10% neutral buffered formalin and in 100% methanol (Merck, Darmstadt, Germany) for 1 minute each. After quick washes in PBS and PBS-T, they were blocked in 1% BSA PBS-T for 1–2 minutes at room temperature. Next, the smears were stained with 2.9 μg/mL 1A4 or 2.3 μg/mL 1D3 purified monoclonal for 1 hour and with 20 μg/mL goat anti-mouse Alexa Fluor 488 (Life Technologies) for 30 minutes in a humid chamber at room temperature, with a brief PBS-T wash after each incubation. Finally, the smears were stained with 0.1% Calcofluor White M2R (Sigma Aldrich) for 5 minutes at room temperature in the dark. The smears were examined on an Olympus BX53 microscope under 400x magnification, and photos were acquired with an Olympus DP21 camera. Photos were taken under a UV filter at 10 millisecond exposure time and under a FITC filter at 167 millisecond exposure time.

### Immunofluorescence Assay on Stool Specimens

Stool specimens collected from subjects, including suspected *E*. *histolytica* cases in Dhaka, Bangladesh, were tested by using a commercial Gal/GalNAc ELISA assay (Techlab, Blacksburg, VA), then archived by freezing at -70°C. For immunofluorescence microscopy using antibody 1A4, frozen stool specimens were allowed to thaw for one hour, then were partially liquefied by the addition of PBS. The suspension was filtered through two-ply cotton gauze to remove large particulates, then smeared onto Merifluor treated slides. Dry smears were fixed in 10% neutral buffered formalin for 1 minute and then stained with primary and secondary antibodies as described above for xenic cultured amoeba. Finally, smears were stained with 1 μg/mL 4',6-diamidino-2-phenylindole (DAPI; ThermoFisher Scientific, Waltham, MA) for 5 minutes at room temperature in the dark. Stained smears were then subjected to a blinded evaluation as follows. Stained smears were examined by light and fluorescence microscopy on a Leica DMLB microscope under 400x magnification, coupled with a Leica DFC310FX digital camera. Three smears per specimen were scanned for cyst-like objects (circular and between ten to twenty micrometers in diameter). The blinded examiner identified the five fields per smear that had cyst-like objects with the brightest visual Jacob2 staining (by 1A4 primary antibody and FITC-labeled secondary antibody). Photos were taken under a FITC filter (Leica 11513808) at an exposure time of 355 milliseconds, under a DAPI filter (Chroma 310000v2) at an exposure time between 1 to 5 milliseconds, and under brightfield. This analysis yielded fifteen images per specimen (3 smears per specimen, 5 images per smear) that had the strongest-staining potential cyst-like objects.

### Image and Statistical Analyses

Each photo taken under the UV filter (Calcofluor or DAPI) was sized to 1300 x 975 pixels on GIMP2.8 and converted to 8-bit grayscale on ImageJ (National Institutes of Health) [28]. Background was subtracted with a 50-pixel rolling ball radius, sliding paraboloid enabled and smoothing disabled. The image was then converted to binary after setting the threshold to ≥12 (for cysts in xenic culture) or ≥19 (for raw stool specimens), and the watershed algorithm was applied to separate clustered particles. Resultant particles that were 1000–5000 square pixels in area and with circularity ranging 0.65–1.00 (holes included, excluded on edge) were labeled as cyst-like objects. Labels on the UV filter photos were transferred to the corresponding FITC filter photos, and the mean “gray value” for each labeled object was obtained as a measure of antibody (1A4 or 1D3) staining. This value is termed “mean fluorescence index” (MFI) in the results.

Statistical analysis was conducted with Microsoft Excel and the Real Statistics Resource Pack software (Release 3.5). The mean gray value of each identified cyst-like object under the FITC filter, a proxy for Alexa Fluor 488 fluorescence intensity, was grouped by isolate. The overall mean fluorescence intensities of the *E*. *histolytica* isolates were compared to *E*. *dispar* isolates and *E*. *bangladeshi* isolates with the Mann-Whitney U test (n = m = 3, α = 0.05). For analyzing antibody staining of cyst-like objects in stool specimens, maximum rather than mean MFI was compared between specimens, because many specimens had few or no cyst like objects, precluding calculation of means. Moreover, a t-test was used to compare the ELISA-positive and ELISA-negative groups.

## Results

### Generation of Jacob2-Specific Antibodies

**Fig 1:**
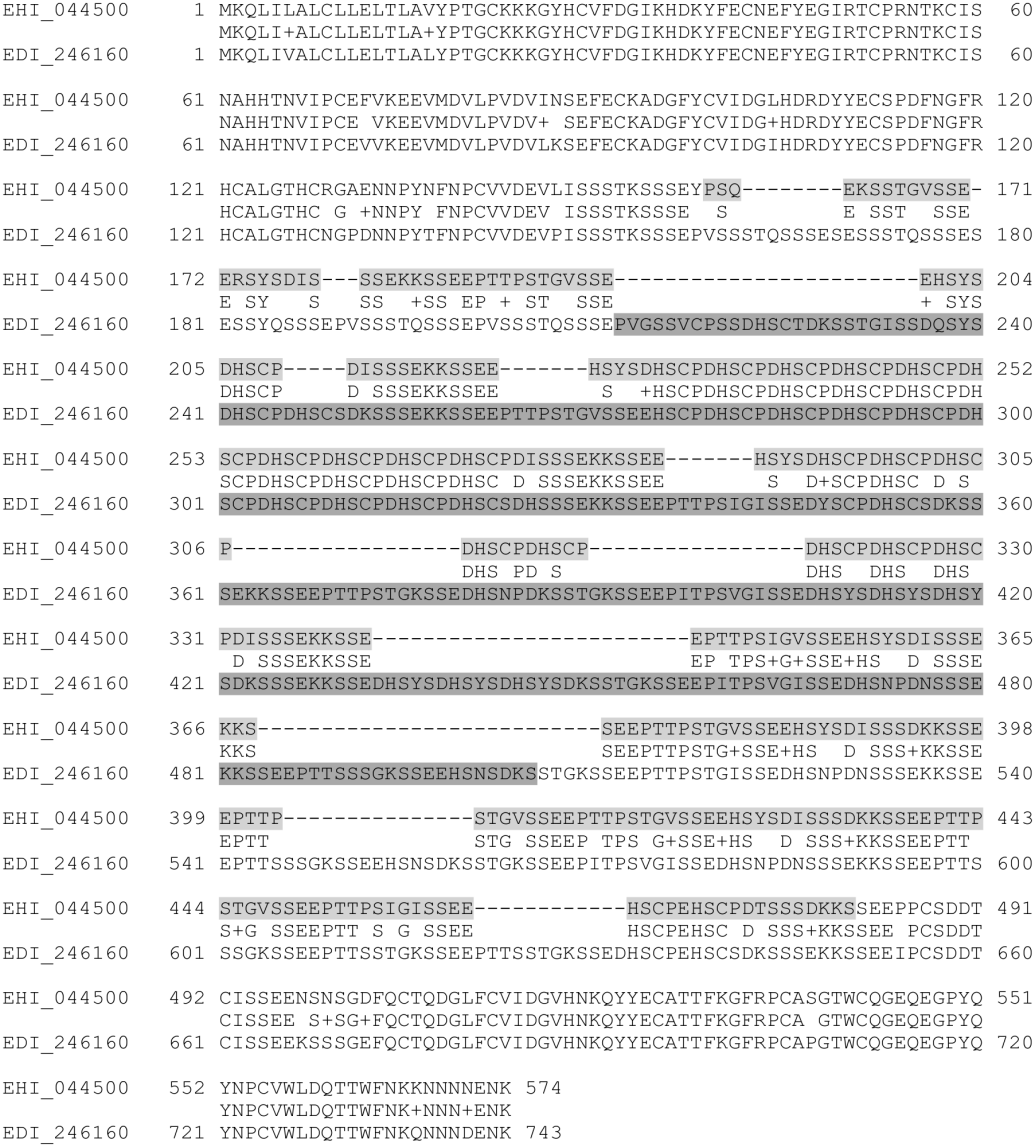
ClustalW alignment of the Jacob2 proteins from *Entamoeba histolytica* HM-1:IMSS (EHI_044500) and *Entamoeba dispar* SAW760 (EDI_246160). The ClustalW BLOSUM matrix was used with a gap open cost of 10 and a gap extension cost of 0.1. This figure was generated in Geneious 6.0.3. Cloned residues for “EhJacob” and “EdJacob” recombinant antigens are highlighted in light gray and dark gray respectively.

The target of this study was the Jacob2 lectin, which was previously shown to be expressed only on the cyst cell wall of *Entamoeba* species. The spacer region of this protein was considered potentially useful as a diagnostic target, because its amino acid sequence diverges between *Entamoeba* species. Fig 1 is a protein sequence alignment of the Jacob2 lectin from *E*. *histolytica* HM-1:IMSS (EhJacob) and *E*. *dispar* SAW760 (EdJacob). The highlighted regions were cloned for expression in this study. The full length-proteins have 64% identity and 22% gaps; however, the cloned regions (which are mostly comprised of the spacer regions) have 53% identity and 15% gaps. The cloned constructs expressed well in BL21(DE3) cells with codon optimization, and high purity protein was obtained with Ni-NTA affinity chromatography (not shown). The SUMO tag was cleaved from EhJacob protein prior to immunization.

EhJacob protein was combined in a cocktail of four immunogens, and immunization and fusion resulted in 108 hybridoma culture supernatants that were specific to EhJacob by indirect ELISA. This pool was narrowed based on activity and specificity, in a stepwise process summarized in Table 1. Out of 108 antibodies that bound to EhJacob in the first step, 48 IgG1 and 16 IgG2 antibodies (N = 64) with the highest activity in indirect ELISA (ODs >1.0) were further screened against both EhJacob and EdJacob by using 48 x 16 matrix sandwich ELISAs. Of these, 41 bound EhJacob antigen as part of at least one pair. However, only 20 did not cross-react with EdJacob protein. Of these, 11 antibodies were examined in a third step involving further indirect ELISA. Six of the 11 clones were eliminated as SUMO-directed antibodies, based on their cross-reaction with an irrelevant, SUMO-tagged protein. This left five antibodies (two IgG1 and three IgG2a) that specifically bound to recombinant EhJacob protein.

**Table 1:** Summary of screens for α-Jacob murine monoclonal antibodies.

					By Isotype		
Screening Step	Screening Assay	Binding Properties	Total Screened	Total Positive (%)	IgG1	IgG2a	IgG2b
1	Indirect ELISA	Binds to EhJacob	1440	108 (7.5%)	78	21	9
2	Sandwich ELISA	Binds to EhJacob	64	41 (64.1%)	29	11	1
		Not Cross-Reactive to EdJacob	64	20 (31.3%)	9	11	0
3	Indirect ELISA	Not Cross-Reactive to SUMO	11	5 (45.5%)	2	3	0

### Limit of Detection in Sandwich Assays

**Fig 2:**
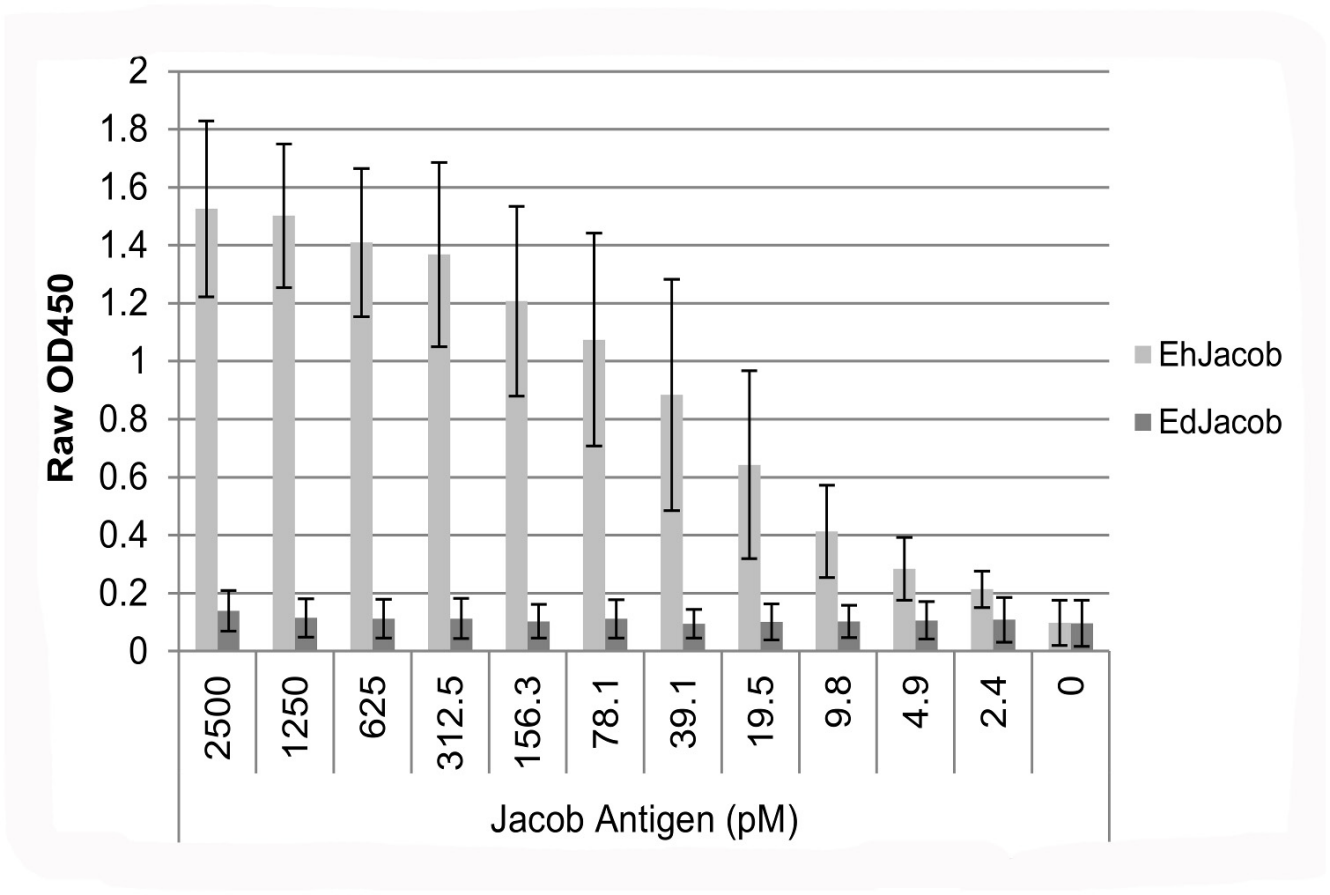
Monoclonal antibodies 1A4 and 1D3 detecting *Entamoeba* recombinant Jacob2 antigens in a sandwich ELISA. Measured antigen concentration ranged from 2.4–2500 pM. Columns and error bars represent mean OD450 ± standard deviation of three replicate assays. Limit of detection (LOD) was calculated as three standard deviations above the mean OD450 of EdJacob at 2500 pM concentration.

Out of the 5 EhJacob-specific antibodies, one IgG1 antibody (designated 1A4) and one IgG2a antibody (designated 1D3) were chosen for further investigation and purified. Their limit of detection as a pair in a sandwich ELISA was determined (Fig 2). When 1A4 capture antibody was eliminated from the sandwich, signals were OD450 < 0.2, indicating that the Jacob antigens did not non-specifically bind to the plate. With 1A4 as the capturing antibody, the two antibodies could detect recombinant *E*. *histolytica* Jacob2 antigen in buffer at concentrations down to 9.8 pM (67 pg). The signal at this antigen concentration was more than three standard deviations greater than the signal with non-cognate EdJacob at 2500 pM concentration.

### Microscopy

**Fig 3:**
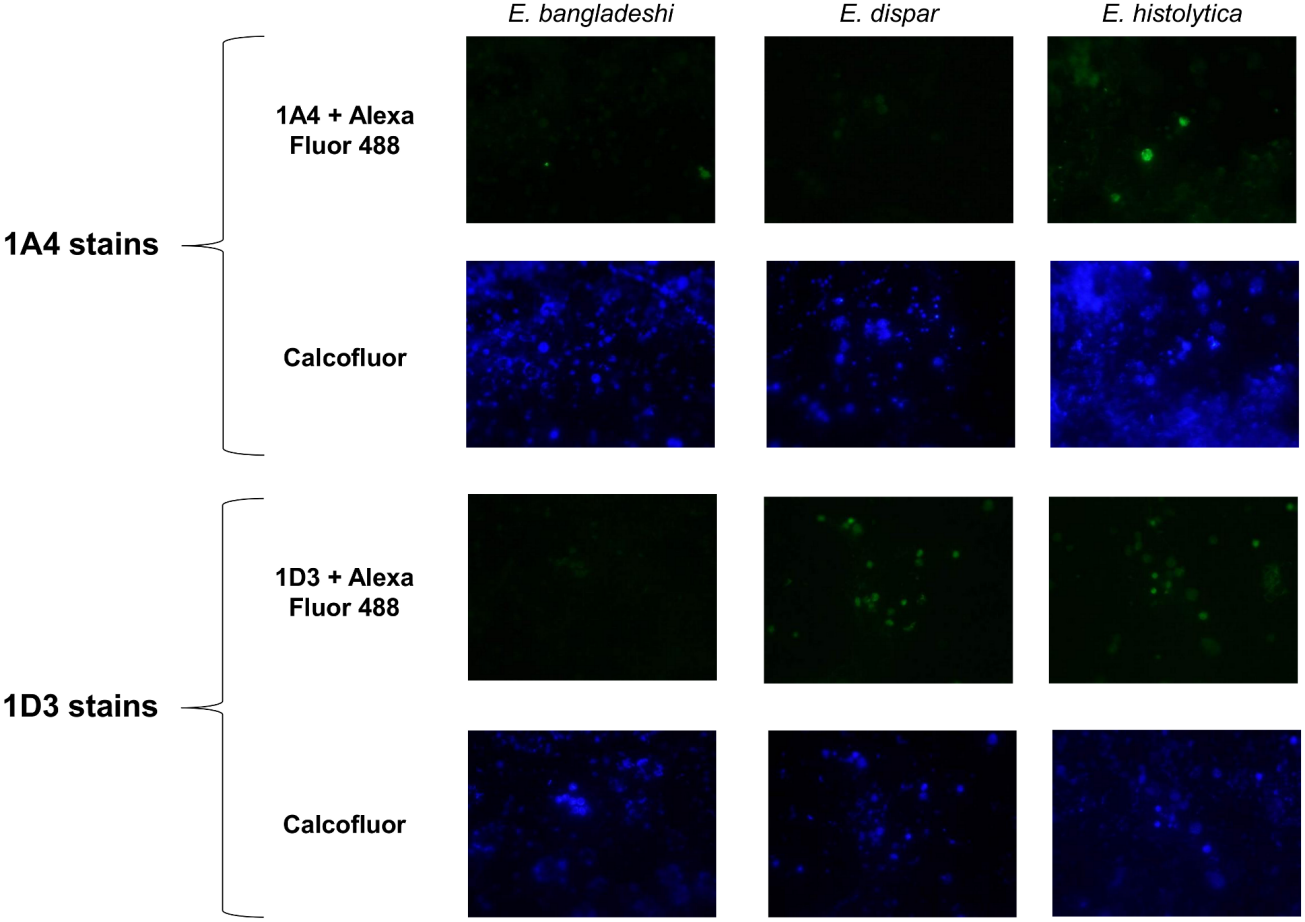
Representative photos from an anti-Jacob2 immunofluorescence microscopy assay. Isolates were doubled stained with 0.1% Calcofluor White M2R and primary anti-Jacob2 monoclonal antibody (1A4 or 1D3) with goat anti-mouse Alexa Fluor 488. The three species examined were pathogen *Entamoeba histolytica* (1^st^ column) and commensals *Entamoeba dispar* and *Entamoeba bangladeshi* (2^nd^ and 3^rd^ columns). Calcofluor was utilized to identify chitinaceous *Entamoeba* cysts (2^nd^ and 4^th^ rows).

Although the 1A4 and 1D3 antibodies exhibited excellent analytical sensitivity and selectivity in an ELISA format, it is not known whether Jacob2 can be detected as a free soluble protein in patient stool specimens. Therefore, we evaluated whether these antibodies can detect whole *Entamoeba* cysts in immunofluorescence microscopy assays conducted on xenic cultures of *E*. *histolytica*, *E*. *dispar*, and *E*. *bangladeshi*. Three independent isolates derived from stool were tested for each species. Xenic culture samples were smeared onto slides, fixed, blocked, and stained with 1A4 or 1D3 antibody, followed by labeling with goat anti-mouse Alexa Fluor 488 secondary. Calcofluor White M2R stain was used as a marker of encystation, as previously established [29]. Fig 3 is a panel of representative photos for each species with each antibody. By eye, IgG1 antibody 1A4 only produced fluorescent cyst-like objects when staining *E*. *histolytica* isolates. In contrast, cross-reaction was seen when Ig2a antibody 1D3 stained *E*. *dispar* isolates.

**Fig 4:**
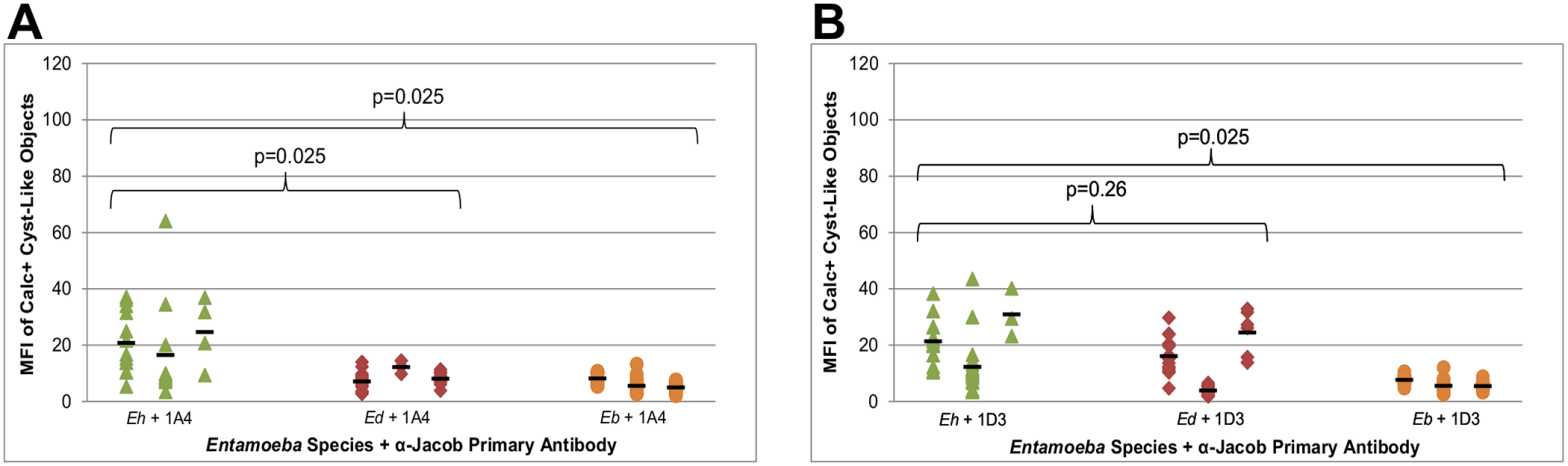
*Entamoeba* species specificities of a-Jacob antibodies 1A4 (A) and 1D3 (B) in immunofluorescence microscopy. Smears of xenic *Entamoeba* isolates were doubly stained with 0.1% Calcofluor White M2R and an anti-Jacob primary antibody with goat anti-mouse Alexa-Fluor 488. Each data point represents the mean Alexa Fluor 488 intensity of a Calcofluor-positive, cyst-like object detected by microscopy. The data points are arranged horizontally by isolate, and black horizontal bars represent the mean Alexa Fluor 488 intensity of each isolate. The overall mean Alexa Fluor 488 intensities of the *Entamoeba histolytica* isolates (n = 3) were compared to those of *E*. *dispar* isolates (n = 3) and of *Entamoeba bangladeshi* isolates (n = 3) with the Mann-Whitney U test. *Eh* = *Entamoeba histolytica*, *Ed = Entamoeba dispar*, *Eb = Entamoeba bangladeshi*.

Computer-assisted image analysis was used to quantify complete observations. Cyst-like objects were identified post-photo acquisition based on Calcofluor intensity, size, and circularity. Mean Alexa Fluor 488 fluorescence intensities of identified cyst-like objects were then calculated. Results are shown in Fig 4 by species and isolate. When measured by a Mann Whitney U hypothesis test (n = m = 3, α = 0.05), the increased mean fluorescence of 1A4-stained *E*. *histolytica* cyst objects over 1A4-stained *E*. *dispar* and *E*. *bangladeshi* cyst objects was statistically significant (one-tailed p-value = 0.025) (Fig 4A). The higher fluorescence of 1D3 stained *E*. *histolytica* objects compared to 1D3-stained *E*. *bangladeshi* was also statistically significant (one-tailed p = 0.025), but the fluorescence of *E*. *histolytica* and *E*. *dispar* objects was not significantly different (one-tailed p = 0.26) (Fig 4B). Two of the three *E*. *dispar* isolates appeared to cross-react with this antibody.

These quantitative results supported the results seen by eye. Although many cyst-like objects did not stain with either antibody regardless of species, strongly-staining objects were confined to *E*. *histolytica* in the case of 1A4 or to *E*. *histolytica* + *E*. *dispar* in the case of 1D3.

Antibody 1A4 was chosen for further evaluation using raw (uncultured) stool specimens collected from subjects in Bangladesh. Frozen specimens were thawed, filtered to remove large particulate matter, applied to microscope slides, formalin-fixed, and analyzed by immunofluorescence microscopy. Methods were similar to those in Fig 4, except the following changes were made to accommodate the much greater complexity of raw stool samples: 1) DAPI was used in place of Calcofluor staining to identify cyst-like objects (DAPI offered better specificity); 2) stained slides were examined by a blinded reader who identified and photographed 15 fields per specimen that contained the most promising 1A4 antibody-stained cyst-like objects; and 3) a more stringent threshold value of 19 was used in image analysis of DAPI-stained cyst-like objects. The sample included ten specimens that had tested positive for *E*. *histolytica* by commercial Gal/GalNAc ELISA (absorbance > 0.1 in ELISA assay), and seven specimens that had tested negative by Gal/GalNAc ELISA (absorbance ≤0.1). Among the ELISA-positive specimens, absorbance values ranged from 0.171 to 1.199. Among the ELISA-negative specimens, the values ranged from 0.032 to 0.057.

**Fig 5:**
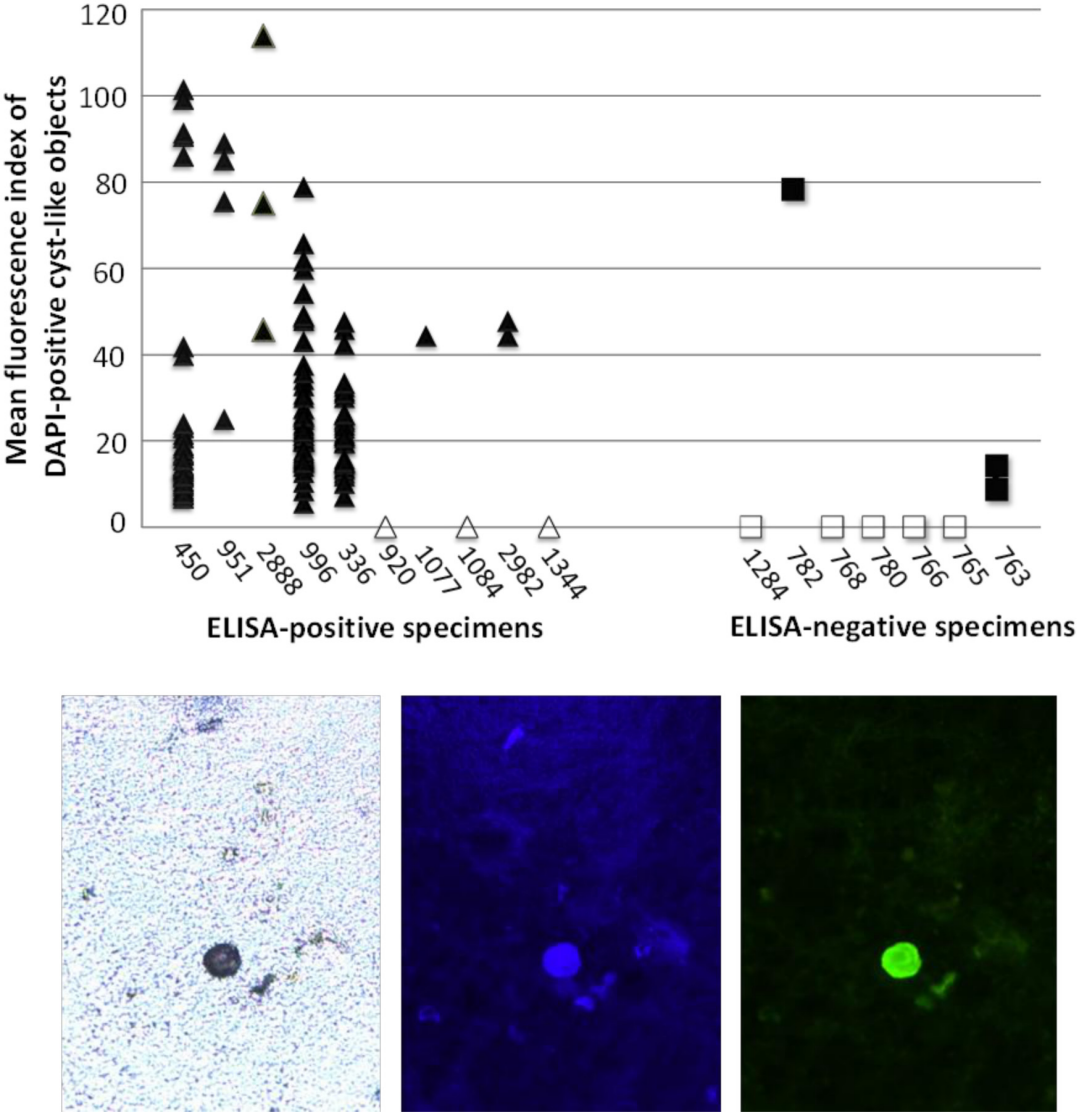
Immunofluorescence staining and image analysis of stool specimens stained with antibody 1A4. ELISA-positive (triangle) and ELISA-negative (square) specimens were stained with DAPI and antibody 1A4 as described in the text. Eight specimens (open symbols) exhibited no detectable cyst-like objects under the DAPI filter. The bottom panels show a typical cyst detectable by (left to right) bright field, DAPI staining, and antibody 1A4 staining microscopy conducted on a fixed stool slide.

For each of the 17 specimens, a visual analyst who was blinded to ELISA results generated 15 photographs containing the cyst-like objects that appeared to stain most strongly with antibody 1A4. These photographs were then subjected to computerized image analysis that first identified all DAPI-staining cyst-like objects, and then quantified mean fluorescence index (MFI) of 1A4 staining (FITC filter) for each such object. Results are shown in Fig 5. Seven of the ten ELISA-positive specimens exhibited at least one DAPI-stained cyst-like object. Each of these specimens included at least a subset of objects that also stained strongly with antibody 1A4 (mean fluorescence index [MFI] > 40). The remaining three ELISA-positive specimens (specimen numbers 920, 1084, and 1344) had no detectable (by DAPI staining and image analysis) cyst-like objects. Among the seven ELISA-negative specimens, five specimens exhibited no detectable cyst-like objects by DAPI staining and image analysis. One ELISA-negative specimen (#782) had a single cyst-like object that stained strongly with antibody 1A4 (MFI = 78). Another ELISA-negative specimen (#763) had two small cyst-like objects that stained very weakly with antibody 1A4 (MFI < 20). S1 Fig shows a visual comparison between one of these weakly-staining objects in specimen #763, and a strongly-staining object (MFI >40) in an ELISA-positive specimen, #951.

Given the small number of cyst-like objects in the ELISA-negative group, statistical comparison of the two groups required that specimens with no cyst-like objects be assigned MFI values of zero. In a one-tailed t-test comparing maximum MFI in the ten ELISA-positive specimens to maximum MFI in the seven ELISA-negative specimens, staining with antibody 1A4 was significantly greater in the ELISA-positive group (p = 0.038). In contrast to Fig 4, this analysis lacked specimens with confirmed cysts of non-pathogen *Entamoeba* species. However, the results demonstrate that antibody 1A4 can be used to stain naturally-occurring *E*. *histolytica* cysts in raw (non-cultured), fixed stool specimens.

## Discussion

This report describes novel antibodies that detect *Entamoeba* cysts. They were generated against the flexible, serine-rich spacer of the Jacob2 lectin, expressed as a recombinant protein fragment in the absence of more conserved portions of Jacob2. Both antibodies were specific to *E*. *histolytica* recombinant antigen, and they detected this antigen with excellent sensitivity in a sandwich ELISA format. However, only the IgG1 antibody designated 1A4 bound whole *E*. *histolytica* cysts in culture without cross-reacting either to *E*. *dispar* or *E*. *bangladeshi* cysts. The reason 1D3 was cross-reactive with *E*. *dispar* when applied to whole cysts (Figs 3 and 4), but not when applied to recombinant EdJacob (Table 1), is not known. It may reflect different physical conditions and availabilities of epitopes in the different assay formats. There could also be a cross-reactive epitope in the native antigen of some *E*. *dispar* isolates. Protein sequence analysis conducted on diverse strains could help resolve this question.

The *E*. *histolytica* Jacob2 lectin drew interest for its spacer divergent from *E*. *dispar* and for its localization on the cyst cell wall [18,19,22]. In this study, the spacer of interest was expressed alone to increase success in finding a species-specific reagent. However, as with other *E*. *histolytica* surface antigens, the spacer has been found to vary by tandem repeats amongst *E*. *histolytica* clinical isolates, and such variation may possibly be found within close phylogenetic groupings [30,31]. In the current investigation, the antibody 1A4 bound to cysts derived from a total of ten separate stool specimens (three in Fig 4 and seven in Fig 5). Thus, based on this limited sample, the antibody has broad specificity for *E*. *histolytica* strains, at least within this geographical region. Three other Gal/GalNAc lectin-positive specimens in Fig 5 exhibited no detectable (by DAPI staining and image analysis) cyst-like objects. Therefore, it could not be confirmed whether or not the *E*. *histolytica* populations in these specimens could be recognized by the 1A4 antibody. Cysts in these specimens may have been present in very low numbers, or they may have been damaged and lost in the freeze-thaw cycle. Given the small and geographically limited sample used in this study, additional research is needed to evaluate whether Jacob2-targeted antibodies can detect a globally useful fraction of *E*. *histolytica* strains. If needed for global coverage, additional antibodies can be generated by the strategy described here. We have generated at least three additional murine antibodies (2C7, 4A4, 7D6) and three yeast-displayed, human single chain fragment variable (scFv) antibodies that could be investigated for this purpose. Despite these limitations, the current study supports the feasibility of designing species-specific antibodies targeted to the Jacob2 spacer region.

In experiments conducted on xenic cultures of non-*histolytica Entamoeba* species, and on Gal/GalNAc-negative stool specimens, the antibody 1A4 exhibited good specificity for *E*. *histolytica*. However, one of the seven Gal/GalNAc ELISA-negative stool specimens contained a single cyst-like object that stained strongly with antibody 1A4. This may represent a false-positive result; however, it is important to note that the reference ELISA method is only ~80% sensitive relative to clinical and molecular (PCR) gold standards (17). Some samples from *E*. *histolytica* patients may contain cysts but relatively few trophozoites, possibly resulting in false-negative readings by Gal/GalNAc ELISA (15,16). Further investigation is needed using larger sample sets and discrepancy resolution methods such as PCR and clinical criteria.

This investigation demonstrated detection of cysts in formalin-fixed samples. Many currently available and reported assays for *E*. *histolytica* detect trophozoites only, or are unable to detect organisms in fixed specimens [9,32]. As trophozoites are unstable outside of the host, this has required that testing be performed on fresh specimens that have been quickly transported to a laboratory [2,9]. The ability of antibody 1A4 to detect *E*. *histolytica* cysts in thawed, formalin-fixed stool smears suggests that the Jacob2 spacer region is a robust target for diagnostics. The ability to test specimens that were fixed upon collection may be a significant logistical advantage in some settings. Another potential advantage of Jacob2 detection is ability to use fluorescence microscopy, a routine procedure in many central and peripheral clinical laboratories.

Despite limitations, this work shows promise toward validating a new biomarker for use in species-specific detection of *E*. *histolytica* cysts. Monoclonal antibody 1A4 is one of few cyst-targeted immunoreagents to distinguish *E*. *histolytica* from nonpathogenic *Entamoeba* species, and, to our knowledge, the only reported reagent to bind fixed *E*. *histolytica* cysts specifically [32]. These findings may ultimately benefit end-users in diagnosis of *E*. *histolytica* infection and in reducing the global burden of diarrheal disease caused by this pathogen.

### Accession Numbers

The Uniprot accession numbers for *E*. *histolytica* and *E*. *dispar* Jacob proteins aligned in this manuscript are C4LT72 and B0EEM4.

## Supporting Information

S1 Fig Immunofluorescence staining of ELISA-positive and ELISA-negative stool specimens stained with antibody 1A4. Top row, left-right: Bright field, DAPI staining, and antibody 1A4 staining microscopy conducted on ELISA-positive sample #951 (mean fluorescence index >40). Bottom row, left-right: Bright field, DAPI staining, and antibody 1A4 staining microscopy conducted on negative sample #763 (mean fluorescence index <20). White arrows show the positions of cyst-like objects detected in DAPI-stained images by image analysis.
(TIFF)
