# Supplementary figures and images for: Species-Specific Immunodetection of an Entamoeba histolytica Cyst Wall Protein
Source: PLoS Negl Trop Dis. 2016 May 6;10(5):e0004697. doi: 10.1371/journal.pntd.0004697 (PMC4859568; doi:10.1371/journal.pntd.0004697)

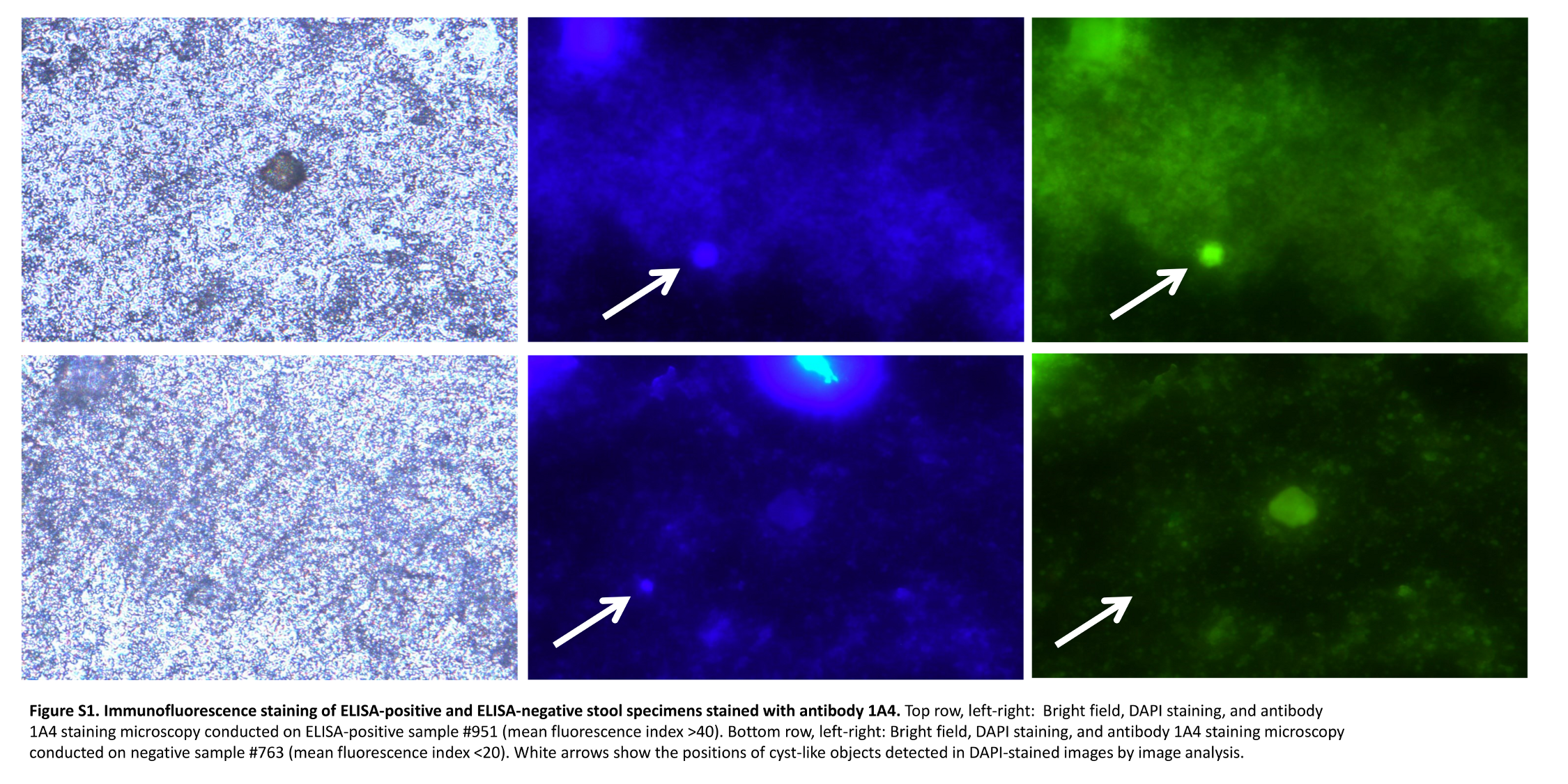

Supplement: S1 Fig — Top row, left-right: Bright field, DAPI staining, and antibody 1A4 staining microscopy conducted on ELISA-positive sample #951 (mean fluorescence index >40). Bottom row, left-right: Bright field, DAPI staining, and antibody 1A4 staining microscopy conducted on negative sample #763 (mean fluorescence index <20). White arrows show the positions of cyst-like objects detected in DAPI-stained images by image analysis. (TIFF) [file pntd.0004697.s001.tiff]
